# Community health volunteers and contraceptive use among adolescent girls and young women in Kenya: a three-wave analysis

**DOI:** 10.1136/bmjph-2024-002368

**Published:** 2025-10-31

**Authors:** William Rudgard, Broline Sagini Asuma, Caroline Kabiru, Chris Desmond, Anthony Idowu Ajayi, Lucie Cluver

**Affiliations:** 1 Social Policy and Intervention, University of Oxford, Oxford, UK; 2 Centre for Social Science Research, University of Cape Town, Cape Town, South Africa; 3 Maseno University, Maseno, Kenya; 4 Sexual, Reproductive, Maternal, Newborn, Child and Adolescent Health (SRMNCAH) Unit, African Population and Health Research Center, Nairobi, Kenya; 5 School of Economics and Finance, University of the Witwatersrand Johannesburg, Johannesburg, South Africa; 6 Department of Psychiatry and Mental Health, University of Cape Town, Cape Town, South Africa

**Keywords:** Adolescent, Sexual Health, Community Health, Social Medicine

## Abstract

**Background:**

Household visits from community health workers can improve adolescent girls and young women’s (AGYW) healthcare seeking and access to services. This study aimed to evaluate whether engagement with community health volunteers (CHVs) is associated with health facility visits, contraceptive use and the fulfilment of family planning needs among AGYW in Kenya.

**Methods:**

We analysed data from three waves of Kenya’s Performance Monitoring for Action cohort, collected between 2019 and 2022. The analysis focused on 3394 AGYW aged 15–24 years who had ever had sex (observations across the three waves=5784). Multivariable mixed-effects unconditional logistic regression was used to evaluate within-individual associations between past-year CHV household visits to talk about family planning and three outcomes: (1) health facility visits; (2) modern contraception use; and (3) unmet need for modern contraception.

**Findings:**

The prevalence of modern contraceptive use and unmet need for family planning among AGYW was 47% and 25%, respectively. 11% were visited by a CHV in the last year. Comparing within individuals across study waves, visits by a CHV increased the likelihood of visiting a health facility (adjusted prevalence ratio (aPR) 1.23, 95% CI 1.14 to 1.31). The combination of a CHV visit and health facility visit showed moderate evidence of increasing the likelihood of modern contraception use (aPR 1.17, 95% CI 1.01 to 1.29), but no evidence of decreasing unmet need for modern contraception (aPR 0.71, 95% CI 0.41 to 1.01).

**Conclusion:**

Household visits by CHVs are likely to connect AGYW to health facilities and support increased contraceptive use. However, it remains unclear if these visits ultimately reduce unmet need for modern contraception among AGYW.

**WHAT IS ALREADY KNOWN ON THIS TOPIC**

- The decline in unmet need for contraception among adolescent girls and young women in Kenya has plateaued. Although the formal role of community health volunteers (CHV) in adolescent sexual and reproductive health is limited in Kenya, there is some evidence suggesting they may help to increase the demand for family planning services.

**WHAT THIS STUDY ADDS**

- Using three waves of the Kenya Performance Monitoring for Action cohort, we find that 11% of adolescent girls who have had sexual intercourse report a home visit by a CHV. These visits are associated with increased healthcare facility use, and the combination of both is associated with increased use of modern contraception, but not with reduced unmet need for contraception.

**HOW THIS STUDY MIGHT AFFECT RESEARCH, PRACTICE OR POLICY**

- This research underscores that CHV outreach may increase contraceptive use among adolescent girls and young women by encouraging visits to health facilities. The lack of evidence for a concurrent decline in unmet need may be because awareness grows faster than access to services. These findings highlight the need to strengthen CHV outreach and enhance support for adolescents seeking contraception at health facilities.

## Introduction

 Adolescent girls and young women (AGYW) aged 15–24 years make up 10% of the Kenyan population.^1^ Improving their access to family planning services and modern contraceptive methods is critical for supporting their reproductive health and autonomy to decide if and when to become pregnant.^2^

In Kenya, significant progress has been made in improving AGYW’s access to family planning. Between 2000 and 2014, the rates of unmet need for contraception among sexually active AGYW nearly halved.^3^ However, since 2014, there has only been a marginal drop in the rates of unmet need for contraception from 35% to 34%. Concurrently, the prevalence of early and unplanned pregnancies has remained stable over the same period at 15%.^4^

Among AGYW, early and unplanned pregnancy is associated with an increased risk of adverse pregnancy outcomes, including unsafe abortion, preterm birth and low birth weight.^5^,^7^ Adolescent mothers also face a higher risk of school dropout and social exclusion from family members, teachers and peers.^8 9^ Improving access to contraception can mitigate these risks with significant economic and social benefits.^10^,^12^

The government of Kenya has made strong commitments to reducing rates of adolescent pregnancy and unmet need for contraception.^13^ The determinants of contraceptive use among AGYW include community norms, contraceptive knowledge, myths and misconceptions, perceived judgement and stigma, and peer use.^14^ There are also supply-side challenges, including inadequate staffing, the cost of services and staff attitudes and discrimination, but evidence also highlights the need to address demand-side barriers.^15^,^17^

In Kenya, community health volunteers (CHVs) play a key role in providing health education and services to families who may not otherwise have access to care nationally.^18 19^ Led and supervised by facility-based community health extension workers, they primarily focus on supporting maternal and child health through intervention strategies including distributing mosquito nets and malaria prophylaxis for pregnant women.^18 20^ They also raise awareness about sexual and reproductive health, offer counselling and health promotion, distribute male condoms and refer girls and women to health facilities for other contraceptive methods.^21^ Although CHVs in Kenya may only distribute a limited range of contraceptive methods, recent evidence from Homa Bay and Narok counties indicates that they may be effective in increasing demand for modern contraception among AGYW.^21^ Further research is needed to validate these findings and determine if increased demand results in higher contraceptive use.

Our aim was to assess the effectiveness of CHVs in increasing AGYW engagement with health services, demand for modern contraceptives and reduction of unmet need for contraception in Kenya.^21^ To achieve this, we analysed if engagement with CHVs is associated with health facility visits, and if the combination of CHV engagement and health facility visits is associated with greater modern contraception use and met need for family planning among AGYW aged 15–24 years in Kenya.

## Methods

This study first describes the prevalence of household visits from CHVs to discuss family planning and, second, uses multivariable regression to assess associations between household visits from CHVs, visiting a health facility, modern contraceptive use and unmet need for modern contraception. Since 2022, CHVs in Kenya are referred to as community health promoters. In our analysis, we use the term ‘CHV’, which was in use during the period in which the study data were collected. The study is reported according to the Strengthening the Reporting of Observational Studies in Epidemiology checklist (online supplemental table S1).^22^

### Study data

The study uses three rounds of ‘Household and Female’ and ‘Service Delivery Point’ (SDP) data from Performance Monitoring for Action (PMA) Kenya panel cohort covering the period from 2019 to 2022.^23^,^25^ Our sample included AGYW aged 15–24 years who had ever had sex. We supplemented individual interview data with SDP data collected from enumeration areas. These included information on the provision and quality of reproductive health services and products, integration of health services, as well as water and sanitation within selected health SDPs in each enumeration area. All data were downloaded from the Integrated Public Use Microdata Series (IPUMS) website.^26^

### Sampling

In Kenya, PMA data collection was implemented by International Centre for Reproductive Health Kenya. A multistage process was used to select participants. 11 out of 47 counties were selected using probability proportional to size in the multistage cluster design.^27^ From the selected counties, 308 enumeration areas were further selected by probability proportional to size after stratifying by urban-rural. After a complete listing of enumeration areas, 35 households were randomly selected for the household survey, and within the selected households, all women of reproductive age (15–49) who consented and were eligible were selected for the female survey. Health SDP data were collected for all public facilities and up to three private facilities within each enumeration area.^27^

### Study variables

*Using modern contraception*. Measured as an aggregated sum of current use of modern contraception methods for either spacing or limiting among all AGYW who had ever had sex. Modern contraceptive methods included emergency contraception, cycle beads, diaphragm, female condoms, female sterilisation, foam jelly, implants, injectable, intrauterine device, lactation amenorrhoea methods, male condoms, male sterilisation and the pill. AGYW using modern contraception were coded as ‘1’, while those who were not were coded as ‘0’.

*Unmet need for modern contraception*. Measured as an aggregated sum of unmet need for spacing and limiting using modern contraception methods among married, fecund and/or sexually active AGYW. AGYW were classified as having unmet need and coded as ‘1’ if they were not using contraception and did not want any more children (limiting); wanted to postpone their next birth for at least 2 years (spacing); or were pregnant or amenorrhoeic and reported the pregnancy or birth as either mistimed or not wanted. AGYW were classified has having met need and coded as ‘0’ if they were using a modern contraceptive method for spacing or limiting. AGYW who had ever had sex, but not in the last 30 days; were not using contraception and desired a child within 2 years; were pregnant or amenorrhoeic and reported the pregnancy or birth as intended; or were infecund were treated as having no unmet need and excluded from the analysis. This categorisation was informed by the approach used by the Demographic and Health Survey (DHS) programme.^28^

*Visit to health facility*. Measured as an indicator of visiting a health facility in the last 12 months. The data were collected through the question: ‘In the last 12 months, have you visited a health facility or camp for care for yourself or your children?’. A response of ‘yes’ was coded as ‘1’, and a response of ‘no’ was coded as ‘0’.

*Household visit from a CHV*. Measured as an indicator of a household visit from a CHV who talked about family planning in the past 12 months. The data were collected through the question: ‘In the last 12 months, were you visited by a community health worker who talked to you about family planning?’. A response of ‘yes’ was coded as ‘1’, and a response of ‘no’ was coded as ‘0’.

*Covariates*. Covariates included nine area-level averages of health SDP characteristics, including (1) number of family planning services offered, (2) number of days per month family planning services and products are offered, (3) percentage with fees for provider consultations, (4) percentage with fees for family planning services, (5) percentage providing family planning counselling to unmarried adolescents, (6) percentage providing family planning services to unmarried adolescents, (7) percentage experiencing contraceptive stock-outs in the last 3 months, (8) percentage that were government owned, and (9) percentage that were hospitals. We also included nine individual-level sociodemographic characteristics, including (1) age, (2) education status measured as highest level of school attended, (3) religious beliefs, (4) marital status, (5) currently pregnant as no and yes, (6) number of live births (parity), (7) location of residence as urban or rural, (8) number of household members and (9) wealth quintile using the same method as the DHS programme,^29^ as well as one indicator of media exposure as a demand-side facilitator for contraceptive use.

### Data analysis

Our analysis had three steps. First, we summarised unweighted characteristics of survey respondents overall and by visit from a CHV. We report count and percentages for categorical variables and median and IQR for continuous variables.

Second, we used regression analysis to evaluate the association between (1) household visits from CHVs and visiting a health facility and (2) how both household visits and health facility visits are associated with modern contraceptive use and separately, unmet need for modern contraception. Our use of longitudinal data enabled us to evaluate how changes in our predictor variables within an individual over time are associated with our outcomes of interest. An advantage of this is that it controls for unobserved, time-invariant differences between individuals that could otherwise confound our estimates of association.

We report both univariable and multivariable associations between predictors and outcomes. Multivariable associations were estimated with unconditional generalised linear mixed-effects model logistic regression specified using the random effects within-between (REWB) modelling framework described in Bell *et al*.^30^ Multivariable models are adjusted for all 19 covariates. All models are specified with two random effects: one that accounts for individuals nested within survey clusters, and another that accounts for repeated observations nested within individuals. We report the amount of variation in study outcomes explained by these random effects. To assess multicollinearity in our regression models, we used the variance inflation factor test.^31^

Third, we used our multivariable models to generate probability trees for modern contraception use and unmet need for contraception conditional on each of the combinations of a household visit from a CHV and visiting a health facility in the last 12 months. We estimated average prevalence ratios and differences by comparing the prevalence of modern contraception use and unmet need for contraception across the different scenarios of receiving a household visit from a CHV and visiting a health facility relative to the reference scenario of neither a CHV visit nor a visit to a health facility.^32^

Our analysis handles missing data by listwise deletion. About 5% of participants were missing data, and therefore we expect this to have minimal influence on our results.

### Patient and public involvement

Informal consultations were conducted with CHVs in Kisumu, Kenya, to contextualise the research and guide the interpretation of findings during data analysis. Patients were not involved in the design, implementation or analysis of this study.

## Results

**Figure 1:**
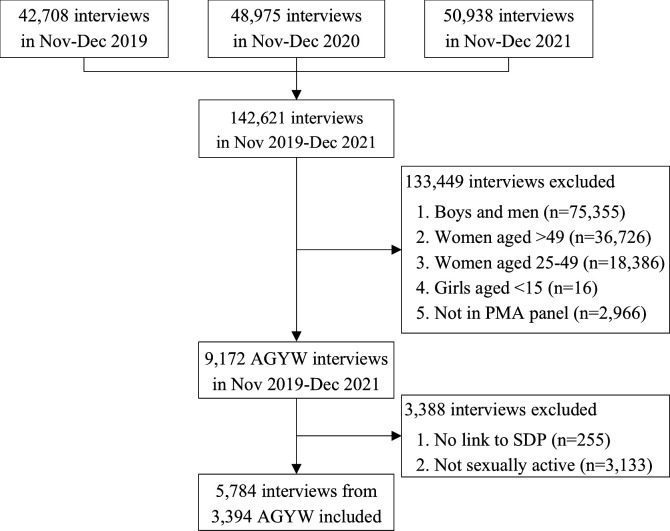
Flow chart of inclusion in our analysis. AGYW, adolescent girls and young women; SDP, service delivery point.

The flow diagram for study inclusion is summarised in figure 1. Across the 3 years, PMA surveyed 34 751 individuals and conducted 142 621 interviews. About 30% of interviews were from the first phase, 34% from the second phase and 36% from the third phase. We excluded 133 449 interviews which were not conducted with AGYW aged 15–24 from the PMA panel sample, 255 that could not be linked to any health SDP data, and 3133 interviews with AGYW who had never had sex. We included data from 5784 interviews with 3394 AGYW. Out of the 3394 participants, 54% were interviewed once (n=1834), 22% twice (n=730) and 24% in all three phases (n=830) (online supplemental table S2). These follow-up patterns reflect our focus on girls who had ever had sex, as well as AGYW mobility and the strong intake of new participants at round 2 to maintain representativeness.^33^

Over the 3-year period, 1054 SDPs were surveyed across 301 enumeration areas. The number of SDPs surveyed in each phase was 999 in phase 1, 993 in phase 2 and 1045 in phase 3, totalling 3037. The mean number of unique SDPs per enumeration area was 6 (SD 0.70). On average, 86% of facilities within enumeration areas were government owned.

### Descriptive analysis

Across three phases, the prevalence of household visits from CHVs was 11%, and slightly over 60% of participants visited a health facility in the 12 months preceding their interview (table 1). The prevalence of modern contraceptive use was 47%, and the prevalence of unmet need for contraception was 25% (table 1). Compared with participants who did not receive a household visit from a CHV, those who did had a higher prevalence of visiting a health facility in the last 12 months (p<0.001) and using modern contraception (p=0.005), while their unmet need for contraception was only slightly lower (p=0.12) (table 1). The most commonly used modern contraceptive methods were implants (17%), injectables (15%) and male condoms (10%), and public sector facilities were the most common source of family planning (86%), as shown in online supplemental tables S3 and S4. SDP and sociodemographic characteristics of participants are also summarised in table 1. AGYW who received a CHV visit were more likely to live in areas with fewer days of family planning service provision, fewer SDPs providing contraceptives to unmarried adolescents, and more SDPs that had experienced recent stock-outs. They were also more likely to be married, to have higher parity, and to reside in rural areas and larger households.

**Table 1:** Unweighted characteristics of AGYW between 2019 and 2022, overall and stratified by visit by a CHV

	Total (n=5784)	Visited by a CHV		P value
		No (n=5155)	Yes (n=627)	
Visited a health facility in the last 12 months				<0.001
No	2137 (37)	1997 (39)	139 (22)	
Yes	3646 (63)	3157 (61)	488 (78)	
Use of any modern contraception				0.005
No	3048 (53)	2748 (54)	299 (48)	
Yes	2693 (47)	2365 (46)	327 (52)	
Unmet need for modern contraception				0.12
Met need	2881 (75)	2531 (75)	349 (78)	
Unmet need	959 (25)	861 (25)	98 (22)	
**SDP characteristics**				
Mean number of FP services offered (per SDP)	5.94 (1.70)	5.94 (1.68)	5.93 (1.86)	0.89
SDPs with fees for FP services (%)	13 (25)	13 (24)	14 (26)	0.73
SDPs with fees to see provider (%)	5 (14)	5 (13)	5 (16)	0.21
Mean number of days per month FP services are offered (per SDP)	19.14 (4.08)	19.17 (3.98)	18.83 (4.80)	0.047
SDPs where unmarried adolescents are counselled on FP (%)	94 (16)	94 (16)	95 (15)	0.39
SDPs where unmarried adolescents are provided with contraceptive methods (%)	93 (18)	93 (17)	91 (22)	0.043
SDPs with any contraceptive stock-out in the last 3 months (%)	18 (27)	18 (26)	22 (32)	<0.001
Government owned SDPs (%)	86 (23)	86 (22)	87 (24)	0.72
SDPs that are hospitals (%)	11 (22)	11 (22)	12 (25)	0.26
**Sociodemographic characteristics**				
Age	20.55 (2.43)	20.53 (2.43)	20.70 (2.38)	0.085
Highest level of school attended				0.25
Never attended	119 (2)	110 (2)	8 (1)	
Primary	1772 (31)	1578 (31)	194 (31)	
Post-primary vocational	155 (3)	142 (3)	13 (2)	
Secondary/A level	2954 (51)	2632 (51)	321 (51)	
College	784 (14)	693 (14)	91 (15)	
Religion				0.003
Catholic	1012 (18)	893 (17)	118 (19)	
Protestant/Christian	4202 (73)	3753 (73)	448 (72)	
Muslim/Islam	151 (3)	124 (2)	27 (4)	
Other	400 (7)	371 (7)	29 (5)	
Marital status				0.044
Married/cohabiting	2422 (42)	2134 (41)	286 (46)	
Single/divorced/widowed	3360 (58)	3019 (59)	341 (54)	
Number of times given birth	0.83 (0.93)	0.82 (0.92)	0.94 (0.97)	0.003
Pregnant now				0.52
No	5295 (92)	4717 (92)	576 (93)	
Yes	464 (8)	418 (8)	46 (7)	
Location of residence				0.021
Urban	1811 (31)	1640 (32)	171 (27)	
Rural	3973 (69)	3515 (68)	4568 (73)	
Number of members in the household	5.21 (2.67)	5.18 (2.66)	5.45 (2.72)	0.017
Wealth quintile				0.25
Lowest quintile	1162 (20)	1033 (23)	128 (20)	
Lower quintile	1200 (21)	1074 (21)	126 (20)	
Middle quintile	1274 (22)	1116 (22)	157 (25)	
Higher quintile	1176 (20)	1051 (20)	125 (20)	
Highest quintile	972 (17)	811 (17)	91 (15)	
Access to media and internet				0.12
No	378 (7)	346 (7)	32 (5)	
Yes	5406 (93)	4809 (93)	595 (95)	
Phase				0.36
1	2181 (38)	1959 (38)	220 (35)	
2	1484 (26)	1316 (26)	168 (27)	
3	2119 (37)	1880 (36)	239 (38)	

For SDP characteristics, values are mean (SD) across enumeration areas, calculated by first averaging the SDP-level value within each enumeration area. Percentages reflect the proportion of SDPs with the characteristic of interest. For all other characteristics, values are n (%).

AGYW, adolescent girls and young women; CHV, community health volunteer; FP, family planning; SDP, service delivery point.

### Regression analysis

Results from our REWB multivariable logistic regression models adjusting for area-level SDP and individual-level sociodemographic covariates are summarised in table 2. Focusing on individual deviation in time-varying predictor variables, within-individual increases in household visits from a CHV were associated with increased odds of visiting a health facility (adjusted OR (aOR) 2.36, 95% CI 1.66 to 3.36). There was also evidence that within-individual increases in health facility visits were associated with increased odds of modern contraception use (aOR 1.49, 95% CI 1.14 to 1.94). No evidence was found that within-individual increases in either household visits from a CHV or health facility visits were associated with unmet need for contraception.

**Table 2:** Multivariable regression results

	Visited a health facility in the last 12 months		Use of any modern contraception		Unmet need for modern contraception	
	**aOR; 95% CI**	**P value**	**aOR; 95% CI**	**P value**	**aOR; 95% CI**	**P value**
Household visit from a CHV						
Within	2.36; 1.66 to 3.36	**<0.001**	1.06; 0.73 to 1.54	0.426	0.74; 0.47 to 1.18	0.207
Between	3.35; 2.31 to 4.86	**<0.001**	1.52; 1.02 to 2.28	**0.042**	0.71; 0.45 to 1.13	0.145
Visited a health facility						
Within			1.49; 1.14 to 1.94	**0.004**	0.85; 0.60 to 1.19	0.341
Between			1.55; 1.20 to 1.99	**0.001**	0.95; 0.71 to 1.26	0.709

The three regression models were based on samples of 5707, 5665, and 3801 observations, respectively. Results with p<0.05 are shown in bold. aORs for area-level service delivery point and individual-level sociodemographic covariates are reported in online supplemental table S5. We report the amount of variation in study outcomes explained by our two random effects in online supplemental table S7 and variance inflation factors for each covariate in online supplemental table S8. Continuous service delivery point characteristics were standardised (mean=0, SD=1). Service delivery point characteristics originally measured as percentages were expressed as proportions (0–1) for regression analysis.

aOR, adjusted OR; CHV, community health volunteer.

Full aORs for area-level and individual-level covariates from the multivariable REWB models are reported in online supplemental table S5. Health facility visits were more common in areas with more SDPs that charged provider fees or provided family planning to adolescents, and less common where more SDPs were government owned. Modern contraceptive use was higher in areas with more government owned SDPs, but lower where more SDPs provided family planning counselling to adolescents. Unmet need was also lower in areas with more government owned SDPs. At the individual level, adolescent girls (15–19 years) had lower odds of modern contraceptive use and higher odds of unmet need compared with women aged 20–24 years. Unmarried girls and women were less likely to visit a health facility or use modern contraception. Higher parity was associated with higher contraceptive use and lower unmet need. Lower education and wealth were associated with lower odds of visiting a health facility or using modern contraceptive, and with higher unmet need.

Results from the univariable regressions are summarised in online supplemental table S6, while online supplemental table S7 reports the proportion of outcome variation explained by the two random effects, and online supplemental table S8 presents the variance inflation factors for each covariate.

### Probability trees

**Figure 2:**
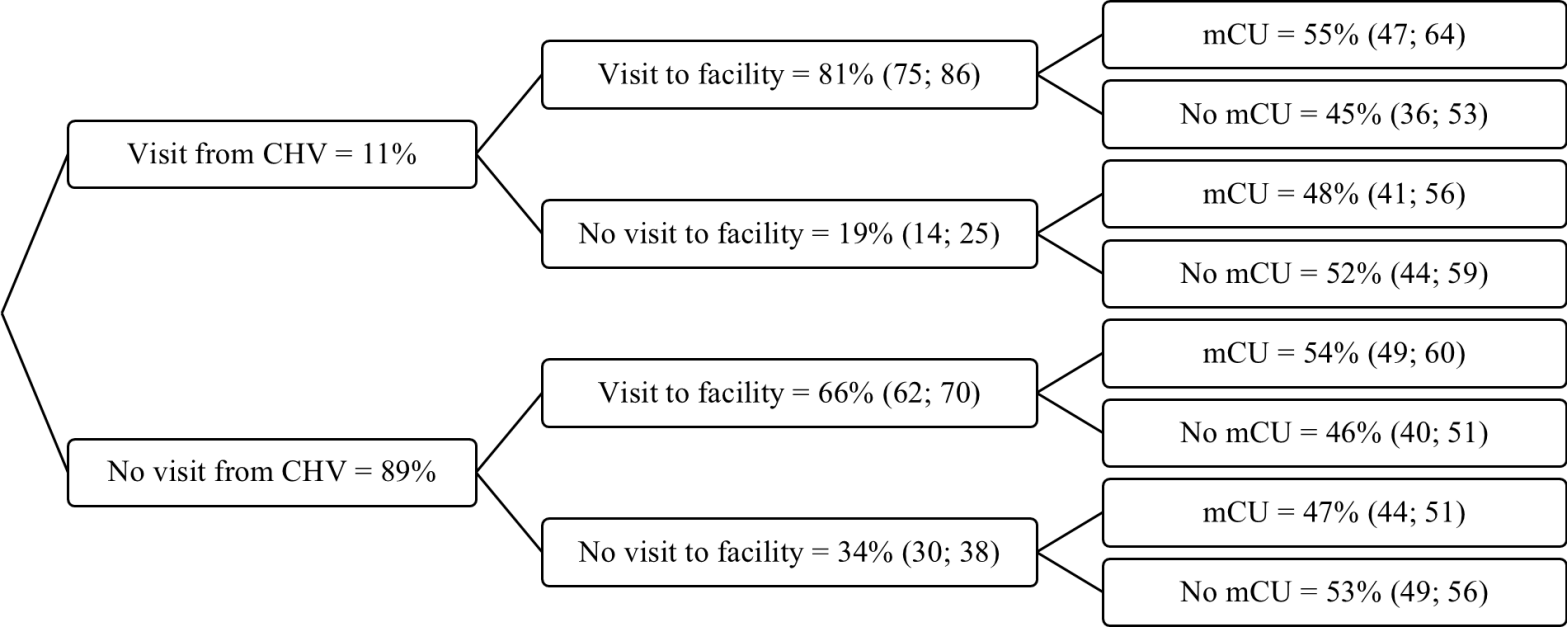
Probability tree for modern contraceptive use, conditional on changes within individuals in household visits from a CHV and visiting a health facility in the last 12 months. CHV, community health volunteer; mCU, modern contraceptive use.

We used probability trees to evaluate how household visits from CHVs and health facility visits combine to influence modern contraceptive use and unmet need for contraception (figures2  3). Within individuals, additional household visits from a CHV were associated with a 23% increase in the probability of visiting a health facility (adjusted prevalence ratio (aPR) 1.23, 95% CI 1.14 to 1.31) (online supplemental table S9). There was also evidence that the combination of additional CHV visits and facility visits was associated with a 17% increase in the probability of modern contraceptive use (aPR 1.17, 95% CI 1.01 to 1.33) (online supplemental table S10). No evidence was found that this combination reduced the probability of unmet need for contraception (aPR 0.71, 95% CI 0.41 to 1.01) (online supplemental table S11).

**Figure 3:**
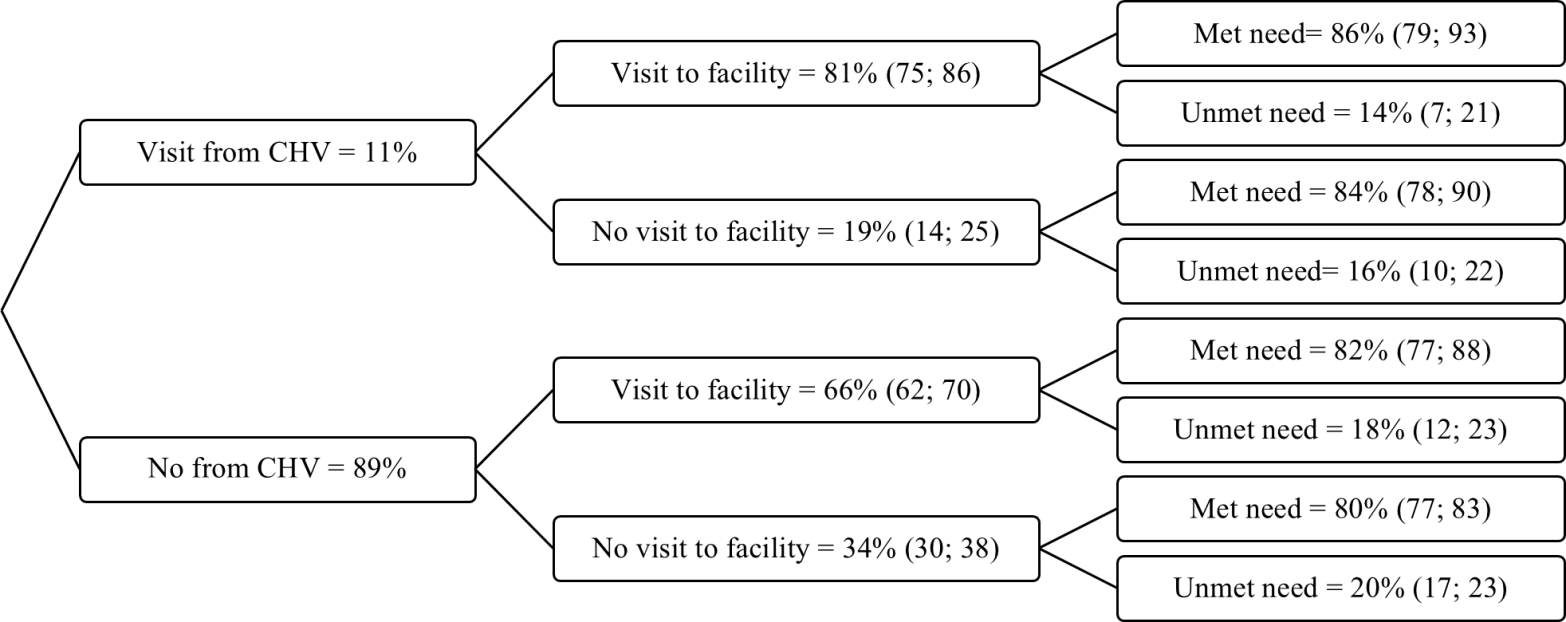
Probability tree for unmet need for modern contraception, conditional on changes within individuals in household visits from a CHV and visiting a health facility in the last 12 months. CHV, community health volunteer.

## Discussion

This analysis investigates the role that CHVs play in promoting contraception use among AGYW in Kenya. We find that while relatively few AGYW receive household visits from CHVs to talk about family planning, those who do are more likely to visit health facilities. Additionally, we find evidence that the combination of both a household visit from a CHV and a visit to a health facility is associated with a higher probability of modern contraceptive use, compared with not receiving either. However, there is no evidence that household visits from a CHV, visits to a health facility or the combination of these factors is associated with lower unmet need for contraception. These patterns suggest that CHV outreach may increase contraceptive use by facilitating health facility visits, while at the same time expanding the pool of those with unmet need, potentially by raising awareness that increases the number of AGYW who desire contraception but are unable to access it.

In considering the prevalence of CHV visits reported by AGYW in our study, previous studies have found that CHVs are likely to take an average of 5 weeks to complete the cycle of households in their catchment area.^34^ This would suggest that most households in our study should have received a visit from a CHV in the last 12 months. Depending on the hours of the day and days of the week that CHVs visit households, CHVs may have missed school-going AGYW.^35^ Alternatively, in some communities, cultural norms may have been a barrier for CHVs discussing family planning with AGYW.^21^

Our result that household visits from CHVs are likely to improve visits to health facilities is consistent with other studies that support the promotive and preventive role of CHVs.^21 36^ The lack of evidence linking CHV visits to unmet need for contraception in our study suggests that additional strategies may be required to achieve the government of Kenya’s ambitious goals for enhancing contraceptive use among adolescents and young adults. Specifically, integrating supply-side interventions with efforts to boost demand for services could be crucial for success.^17^ Addressing key supply-side barriers, such as health facility staff attitudes and knowledge, is one promising avenue for improving outcomes.^16 37 38^

Our study used data collected in three phases of the PMA study, which employed rigorous statistical sampling methods to ensure representative results. We applied advanced statistical methods to analyse within-individual variation in predictors, which is robust to confounding from time-invariant factors. The study also had limitations. Attrition between waves influenced by the high mobility of AGYW may have introduced bias if those lost to follow-up differed systematically from those retained. There is still a risk of confounding from unmeasured time-varying factors for within-individual associations, and estimated associations should not be interpreted causally. We also relied on self-reported data from adolescents and facility heads, which may introduce measurement error due to recall bias.

Acknowledging these strengths and limitations, our study provides valuable insights into the national prevalence and influence of household visits from CHVs on meeting contraceptive needs among AGYW in Kenya. Our findings underscore the necessity of further supporting CHVs to ensure that they discuss family planning with adolescents and young adults during their household visits. There is evidence that efforts to empower CHVs, through remuneration and incentives, and build their capacity around age-appropriate communication techniques can be effective for this.^2139^,^42^

The lack of evidence linking CHV visits to meeting contraceptive needs presents a significant area for future research. Given that pharmacies and shops are often AGYW’s first source of contraception, future studies should explore whether CHVs can increase their use through household visits.^43^ Effective support for young people’s use of sexual and reproductive health services often requires a dual approach that addresses both demand and supply-side barriers.^17^ Future studies should also evaluate whether the effects of CHV household visits depend on how responsive health facilities are to the needs of young people.

In conclusion, household visits by CHVs help connect AGYW to health facilities and contribute to improving modern contraceptive use. However, unmet need remains unchanged, possibly because awareness grows faster than access. Further research is needed to evaluate how CHV outreach, combined with improved facility services, can better meet AGYW’s family planning needs.

## Supplementary material

10.1136/bmjph-2024-002368 online supplemental file 1

## Data Availability

Data are available in a public, open access repository.
