# Supplementary material for: Community health volunteers and contraceptive use among adolescent girls and young women in Kenya: a three-wave analysis
Source: BMJ Public Health. 2025 Oct 31;3(2):e002368. doi: 10.1136/bmjph-2024-002368 (PMC12581035; doi:10.1136/bmjph-2024-002368)
Supplement: online supplemental file 1 [file bmjph-3-2-s001.pdf]

SUPPLEMENTARY APPENDIX

Table S1. STROBE checklist for cohort studies.

	Item No.	Recommendation	Page No.
**Title and abstract**	1	(*a*) Indicate the study's design with a commonly used term in the title or the abstract	1
		(*b*) Provide in the abstract an informative and balanced summary of what was done and what was found	2
**Introduction**			
Background/rationale	2	Explain the scientific background and rationale for the investigation being reported	5
Objectives	3	State specific objectives, including any prespecified hypotheses	5
**Methods**			
Study design	4	Present key elements of study design early in the paper	5
Setting	5	Describe the setting, locations, and relevant dates, including periods of recruitment, exposure, follow-up, and data collection	5
Participants	6	Give the eligibility criteria, and the sources and methods of selection of participants. Describe methods of follow-up	5
Variables	7	Clearly define all outcomes, exposures, predictors, potential confounders, and effect modifiers. Give diagnostic criteria, if applicable	5
Data sources/ measurement	8	For each variable of interest, give sources of data and details of methods of assessment (measurement). Describe comparability of assessment methods if there is more than one group	5
Bias	9	Describe any efforts to address potential sources of bias	6/7
Study size	10	Explain how the study size was arrived at	5
Quantitative variables	11	Explain how quantitative variables were handled in the analyses. If applicable, describe which groupings were chosen and why	5/6
Statistical methods	12	(a) Describe all statistical methods, including those used to control for confounding	6/7
		(b) Describe any methods used to examine subgroups and interactions	6/7
		(c) Explain how missing data were addressed	6/7
		(d) If applicable, explain how loss to follow-up was addressed	-
		(e) Describe any sensitivity analyses	6/7
**Results**			
Participants	13	(a) Report numbers of individuals at each stage of study—eg numbers potentially eligible, examined for eligibility, confirmed eligible, included in the study, completing follow-up, and analysed	8
		(b) Give reasons for non-participation at each stage	8
		(c) Consider use of a flow diagram	8
Descriptive data	14	(a) Give characteristics of study participants (eg demographic, clinical, social) and information on exposures and potential confounders	8
		(b) Indicate number of participants with missing data for each variable of interest	10
		(c) Summarise follow-up time (eg, average and total amount)	-
Outcome data	15	Report numbers of outcome events or summary measures over time	-
Main results	16	(a) Give unadjusted estimates and, if applicable, confounder-adjusted estimates and their precision (eg, 95% confidence interval). Make clear which confounders were adjusted for and why they were included	11
		(b) Report category boundaries when continuous variables were categorized	10
		(c) If relevant, consider translating estimates of relative risk into absolute risk for a meaningful time period	-
Other analyses	17	Report other analyses done—e.g. analyses of subgroups and interactions, and sensitivity analyses	9
**Discussion**			

Key results	18	Summarise key results with reference to study objectives	13
Limitations	19	Discuss limitations of the study, taking into account sources of potential bias or imprecision. Discuss both direction and magnitude of any potential bias	13/14
Interpretation	20	Give a cautious overall interpretation of results considering objectives, limitations, multiplicity of analyses, results from similar studies, and other relevant evidence	13/14
Generalisability	21	Discuss the generalisability (external validity) of the study results	13/14

Table S2. Rates of individual follow-up patterns across the three waves of data collection.

First follow-up pattern	N (%)	Second follow-up pattern	N (%)
10	968 (28)	100	843 (25)
		101	125 (4)
11	1213 (36)	110	383 (11)
		111	830 (24)
01	271 (8)	010	49 (1)
		011	222 (7)
00	942 (28)	001	942 (28)

Table S3. Contraceptive method mix among sexually active adolescent girls and young women, overall and disaggregated by age-group.

Contraceptive method	Total (N=5,784)	15-19 (N=1,960)	20-24 (N=3,824)	p-value
Implant	970 (17)	179 (9)	791 (21)	<0.001
Injectables	888 (15)	134 (7)	754 (20)	<0.001
Condoms	579 (10)	276 (14)	303 (8)	<0.001
The pill	193 (3)	43 (2)	150 (4)	<0.001
Emergency contraception	53 (1)	14 (1)	39 (1)	0.31
IUD	29 (1)	6 (0)	23 (1)	0.17
Female sterilization	11 (0.19)	2 (0.10)	9 (0.24)	0.35
Male sterilization	9 (0.16)	2 (0.10)	7 (0.18)	0.73
Female condoms	7 (0.12)	2 (0.10)	5 (0.13)	>0.999
Diaphragm	1 (0.02)	0 (0.00)	1 (0.03)	>0.999
Standard days method	30 (0.52)	8 (0.41)	22 (0.58)	0.45
LAM	54 (0.93)	9 (0.46)	45 (1.18)	0.006
Rhythm method	183 (3.16)	57 (2.91)	126 (3.30)	0.48
Withdrawal	80 (1.38)	18 (0.92)	62 (1.62)	0.032
Other traditional methods	6 (0.10)	0 (0.00)	6 (0.16)	0.10

Values are n (%). Abbreviations: IUD, intrauterine device; LAM, lactational amenorrhea method.

Table S4. Sources of family planning among sexually active adolescent girls and young women reporting using contraception.

Source of family planning	Total (N=2,884)
Public sector	2,478 (86)
Government hospital/ polyclinic	
Government health center	
Government health post	
Family planning clinc	
Mobile clinic	
Fieldworker/ outreach/ peer educator	
Community event	
Private medical sector	197 (7)
Private hospital/ clinic	
Private doctor	
Pharmacy	
Chemical/ drug store	
FP clinic	
Maternity home	
Community event	
Other	209 (7)
Shop/ market	
Church	
Community volunteer	
Friend / relative	

Values are n (%) of all respondents reporting a source of family planning. Abbreviations: FP, family planning.

Table S5. Adjusted odds ratios for area- and individual-level covariates from multivariable regression models.

	**Visited a health facility in last 12 months**		**Use of any modern contraception**		**Unmet need for modern contraception**	
	**aOR; 95%CI**	**p-value**	**aOR; 95%CI**	**p-value**	**aOR; 95%CI**	**p-value**
**Service delivery point characteristics**						
Number of FP services offered (std.)	0.98; 0.87-1.11	0.769	0.87; 0.76-1.00	0.050	1.12; 0.95-1.31	0.177
SDPs with fees for FP services	0.89; 0.55-1.45	0.645	1.17; 0.67-2.05	0.578	0.77; 0.41-1.46	0.430
SDPs with fees to see provider	2.49; 1.39-4.44	**0.002**	0.96; 0.51-1.82	0.910	1.45; 0.70-3.01	0.323
Number of days per month FP services are offered (std.)	1.01; 0.91-1.11	0.916	1.04; 0.93-1.18	0.474	1.02; 0.89-1.17	0.746
SDPs where unmarried adolescents are counselled on FP	0.89; 0.53-1.49	0.655	0.49; 0.27-0.88	**0.018**	1.75; 0.86-3.56	0.123
SDPs where unmarried adolescents are provided with contraceptive methods	1.91; 1.20-3.05	**0.006**	1.42; 0.83-2.43	0.197	0.95; 0.50-1.80	0.869
SDPs with any contraceptive stock out in the last three months	0.93; 0.70-1.22	0.585	1.26; 0.92-1.73	0.155	1.09; 0.74-1.58	0.672
Government owned SDPs	0.37; 0.21-0.64	**<0.001**	2.25; 1.18-4.26	**0.013**	0.42; 0.20-0.88	**0.022**
SDPs that are hospitals	1.12; 0.73-1.74	0.600	0.66; 0.39-1.10	0.110	1.62; 0.91-2.89	0.098

**Sociodemographic characteristics**						
Age category						
15-19	1.09; 0.90-1.32	0.389	0.57; 0.45-0.71	**<0.001**	1.96; 1.51-2.55	**<0.001**
20-24	Ref		Ref		Ref	
Highest level of school attended						
Never attended	0.68; 0.35-1.30	0.238	0.06; 0.02-0.16	**<0.001**	8.56; 3.00-24.37	**<0.001**
Primary	0.58; 0.43-0.78	**<0.001**	1.00; 0.70-1.42	0.988	1.53; 1.01-2.31	**0.044**
Post-primary vocational	0.92; 0.53-1.60	0.760	1.08; 0.57-2.04	0.819	0.97; 0.46-2.03	0.933
Secondary/ A level	0.85; 0.66-1.10	0.220	1.01; 0.76-1.35	0.937	1.13; 0.79-1.62	0.492
Tertiary	Ref		Ref		Ref	
Religion						
Catholic	Ref		Ref		Ref	
Protestant / Christian	0.98; 0.80-1.21	0.871	1.27; 0.99-1.62	0.055	0.72; 0.55-0.96	**0.023**
Muslim / Islam	0.75; 0.43-1.29	0.293	1.28; 0.66-2.45	0.463	0.69; 0.33-1.43	0.317
Other	0.88; 0.62-1.24	0.455	0.90; 0.59-1.37	0.635	1.47; 0.93-2.30	0.098
Marital Status						
Married / cohabiting	Ref		Ref		Ref	
Single / divorced / widowed	0.59; 0.47-0.72	**<0.001**	0.41; 0.32-0.53	**<0.001**	0.83; 0.63-1.10	0.189
Number of times given birth	1.92; 1.69-2.18	**<0.001**	1.76; 1.52-2.03	**<0.001**	0.59; 0.49-0.70	**<0.001**
Pregnant now						
No	Ref		Ref			
Yes	2.05; 1.53-2.75	**<0.001**	0.00; 0.00-0.00	**<0.001**		
Location of residence						
Urban	Ref		Ref		Ref	
Rural	1.45; 0.99-2.12	0.057	0.77; 0.52-1.14	0.190	1.23; 0.82-1.86	0.317
Number of members in household	1.01; 0.98-1.04	0.609	0.96; 0.92-1.00	**0.034**	1.06; 1.01-1.10	**0.009**
Wealth quintile						
Lowest quintile	0.70; 0.51-0.95	**0.025**	0.58; 0.40-0.84	**0.004**	2.21; 1.45-3.38	**<0.001**
Lower quintile	0.94; 0.71-1.25	0.664	0.92; 0.67-1.28	0.635	1.52; 1.04-2.24	**0.032**
Middle quintile	0.87; 0.67-1.13	0.307	1.07; 0.79-1.44	0.669	0.96; 0.66-1.39	0.833
Higher quintile	0.80; 0.63-1.03	0.086	1.02; 0.76-1.35	0.909	1.17; 0.82-1.66	0.399
Highest quintile	Ref		Ref		Ref	
Access to media & internet						
No	0.63; 0.46-0.87	**0.005**	0.73; 0.49-1.09	0.127	1.19; 0.76-1.85	0.455
Yes	Ref		Ref		Ref	

Continuous service delivery point characteristics were standardised (mean = 0, SD = 1). Service delivery point characteristics originally measured as percentages were expressed as proportions (0–1) for regression analysis. Results with p<0.05 are shown in bold. Abbreviations: aOR, adjusted odds ratio; CI, confidence interval; FP, family planning; CHV, community health volunteer; SDP, service delivery point.

Table S6. Univariable regression results.

	Visited a health facility in last 12 months		Use of any modern contraception		Unmet need for modern contraception	
	**cOR; 95%CI**	**p-value**	**cOR; 95%CI**	**p-value**	**cOR; 95%CI**	**p-value**
Household visit from a CHV						
Within	2.18; 1.56-3.06	<0.001	1.30; 0.94-1.79	0.107	0.75; 0.49-1.16	0.198
Between	3.86; 2.65-5.61	<0.001	1.74; 1.22-2.47	0.002	0.60; 0.39-0.93	0.021
Visited a health facility			1.47; 1.16-1.86	0.001	0.79; 0.57-1.09	0.150
Within			1.99; 1.60-2.46	<0.001	0.72; 0.56-0.94	0.016
Between	2.18; 1.56-3.06	<0.001	1.30; 0.94-1.79	0.107	0.75; 0.49-1.16	0.198
**Service delivery point characteristics**						
Number of FP services offered (std.)	0.97; 0.89-1.06	0.516	0.97; 0.89-1.06	0.507	1.06; 0.95-1.18	0.293
SDPs with fees for FP services	1.54; 1.09-2.17	0.014	0.87; 0.62-1.24	0.445	1.07; 0.69-1.66	0.750
SDPs with fees to see provider	2.18; 1.24-3.82	0.007	0.87; 0.50-1.49	0.600	1.66; 0.84-3.31	0.145
Number of days per month FP services are offered (std.)	1.01; 0.94-1.10	0.730	1.04; 0.96-1.13	0.374	1.02; 0.92-1.13	0.738
SDPs where unmarried adolescents are counselled on FP	0.82; 0.51-1.30	0.391	0.62; 0.39-1.00	0.049	1.58; 0.85-2.97	0.151
SDPs where unmarried adolescents are provided with contraceptive methods	1.29; 0.84-1.97	0.242	1.07; 0.70-1.65	0.748	1.07; 0.61-1.88	0.821
SDPs with any contraceptive stock out in the last three months	1.06; 0.81-1.40	0.652	1.14; 0.87-1.50	0.350	1.03; 0.72-1.47	0.879
Government owned SDPs	0.50; 0.35-0.74	<0.001	1.32; 0.90-1.95	0.158	0.82; 0.51-1.32	0.409
SDPs that are hospitals	1.17; 0.80-1.72	0.417	0.75; 0.50-1.11	0.150	1.39; 0.86-2.24	0.175
**Sociodemographic characteristics**						
Age category						
15-19	0.55; 0.47-0.65	<0.001	0.34; 0.28-0.40	<0.001	2.84; 2.26-3.57	<0.001
20-24	Ref		Ref		Ref	
Highest level of school attended						
Never attended	1.44; 0.77-2.68	0.252	0.17; 0.07-0.40	<0.001	7.44; 2.83-19.55	<0.001
Primary	1.11; 0.85-1.45	0.431	1.43; 1.09-1.87	0.010	1.56; 1.10-2.21	0.013
Post-primary vocational	1.54; 0.89-2.65	0.121	1.14; 0.67-1.94	0.631	0.98; 0.49-1.97	0.962
Secondary A level	0.92; 0.73-1.17	0.514	0.94; 0.74-1.20	0.625	1.48; 1.07-2.06	0.018
Tertiary	Ref		Ref		Ref	
Religion						
Catholic	Ref		Ref		Ref	
Protestant / Christian	1.03; 0.83-1.27	0.802	1.30; 1.05-1.61	0.018	0.73; 0.56-0.96	0.024
Muslim / Islam	0.86; 0.49-1.49	0.578	1.26; 0.71-2.25	0.430	0.72; 0.36-1.46	0.365
Other	0.92; 0.65-1.29	0.620	0.94; 0.66-1.35	0.743	1.56; 1.01-2.40	0.044
Marital Status						
Married / cohabiting	Ref		Ref		Ref	
Single / divorced / widowed	0.35; 0.30-0.42	<0.001	0.37; 0.31-0.44	<0.001	1.61; 1.31-1.98	<0.001
Number of times given birth	1.97; 1.78-2.17	<0.001	2.42; 2.17-2.70	<0.001	0.61; 0.53-0.69	<0.001
Pregnant now						
No	Ref		Ref			
Yes	2.00; 1.51-2.64	<0.001	0.00; 0.00-0.01	<0.001		
Location of residence						
Urban	Ref		Ref		Ref	
Rural	1.31; 0.88-1.94	0.185	0.66; 0.44-0.99	0.046	1.63; 0.99-2.67	0.055
Number of members in household	0.98; 0.95-1.01	0.193	0.96; 0.93-0.99	0.010	1.06; 1.02-1.10	0.005
Wealth quintile						

Lowest quintile	0.84; 0.63-1.12	0.243	0.77; 0.57-1.04	0.093	2.23; 1.54-3.24	<0.001
Lower quintile	1.08; 0.82-1.42	0.579	1.10; 0.84-1.46	0.489	1.52; 1.07-2.16	0.020
Middle quintile	0.97; 0.75-1.26	0.823	1.23; 0.95-1.60	0.114	0.93; 0.66-1.31	0.671
Higher quintile	0.89; 0.69-1.14	0.353	1.10; 0.86-1.42	0.443	1.12; 0.80-1.57	0.503
Highest quintile	Ref		Ref		Ref	
Access to media & internet						
No	0.60; 0.45-0.82	0.001	0.67; 0.48-0.93	0.016	1.64; 1.10-2.44	0.015
Yes	Ref		Ref		Ref	

Continuous service delivery point characteristics were standardised (mean = 0, SD = 1). Service delivery point characteristics originally measured as percentages were expressed as proportions (0–1) for regression analysis.Abbreviations: cOR, crude odds ratio; CI, confidence interval; FP, family planning; CHV, community health volunteer.

Table S7. Summary of random effects in the multivariable regression analysis.

	**Visited a health facility in last 12 months**		**Use of any modern contraception**		**Unmet need for modern contraception**	
	**OR; 95% CI**	**p-value**	**OR; 95% CI**	**p-value**	**OR; 95% CI**	**p-value**
Average null model	1.98; 1.61-2.43	**<0.001**	0.91; 0.73-1.13	0.393	0.21; 0.15-0.28	**<0.001**
Random effects by strata	0.18; 0.09-0.38		0.22; 0.11-0.44		0.32; 0.15-0.66	
Random effects strata>subject	2.00; 1.57-2.56		2.41; 1.93-3.03		1.77; 1.17-2.72	

Abbreviations: OR, odds ratio; CI, confidence interval.

Table S8. Variance inflation factor and tolerance in the multivariable regression analysis.

	**Visited a health facility in last 12 months**		**Use of any modern contraception**		**Unmet need for modern contraception**	
	**VIF**	**Tolerance**	**VIF**	**Tolerance**	**VIF**	**Tolerance**
Within; Household visit from a CHV	1.00	0.9969	1.01	0.9925	1.01	0.9931
Between; Household visit from a CHV	1.01	0.9855	1.03	0.9725	1.03	0.9723
Between; Visit to a health facility			1.01	0.993	1.01	0.9929
Within; Visit to a health facility			1.15	0.8674	1.15	0.8723
**Service delivery point characteristics**						
Number of FP services offered (std.)	1.70	0.5871	1.70	0.5867	1.70	0.5868
SDPs with fees for FP services	2.33	0.4284	2.33	0.4284	2.33	0.4283
SDPs with fees to see provider	1.04	0.9615	1.04	0.9601	1.04	0.9604
Number of days per month FP services are offered (std.)	1.42	0.7037	1.42	0.7033	1.42	0.703
SDPs where unmarried adolescents are counselled on FP	1.26	0.7912	1.27	0.7897	1.27	0.7897
SDPs where unmarried adolescents are provided with contraceptive methods	1.22	0.8217	1.22	0.8213	1.22	0.8214
SDPs with any contraceptive stock out in the last three months	1.05	0.9549	1.05	0.9548	1.05	0.9553
Government owned SDPs	2.25	0.4438	2.26	0.4426	2.26	0.4425
SDPs that are hospitals	1.39	0.7183	1.39	0.7183	1.39	0.7186
**Sociodemographic characteristics**						
Age category	1.61	0.6227	1.61	0.6212	1.61	0.6226
Highest level of school attended	1.41	0.7105	1.41	0.7091	1.41	0.7101
Religion	1.04	0.9588	1.04	0.9577	1.04	0.9579

Marital Status	2.11	0.4737	2.13	0.4699	2.01	0.4986
Number of times given birth	2.15	0.4647	2.19	0.4562	2.14	0.4684
Pregnant now	1.09	0.9151	1.1	0.9099		
Location of residence	1.3	0.7706	1.3	0.7675	1.3	0.7678
Number of members in household	1.15	0.8676	1.15	0.8675	1.15	0.8673
Wealth quintile	1.48	0.674	1.48	0.6738	1.48	0.6745
Access to media & internet	1.13	0.8825	1.14	0.8798	1.14	0.8793
Mean VIF	1.44		1.41		1.42	

Abbreviations: VIF, variance inflation factor; CHV, community health volunteers; FP, family planning.

Table S9. Adjusted probabilities of outcomes by experience of household visits from a CHV. Prevalence ratio and prevalence difference compare different combinations of these exposures to a baseline of experiencing no visit from a CHV.

		PP	aPD (95% CI)	aPR (95% CI)
Visiting a facility	No visit by CHV	65.76	Ref	Ref
	Visit by CHV	80.63	14.87 (9.70-20.03)	1.23 (1.14-1.31)
Modern contraceptive use	No visit by CHV	47.42	Ref	Ref
	Visit by CHV	48.49	1.07 (–5.50-7.64)	1.02 (0.88-1.16)
Unmet need for modern contraception	No visit by CHV	19.78	Ref	Ref
	Visit by CHV	15.88	–3.90 (–9.47-1.68)	0.80 (0.52-1.08)

Abbreviations: PP, predicted prevalence, aPR, adjusted prevalence ratio; PD, adjusted prevalence difference; CI, confidence intervals; CHV, community health volunteer.

Table S10. Adjusted probabilities of modern contraceptive use by experience of household visits from a CHV and visit to a health facility. Prevalence ratio and prevalence difference compare different combinations of these exposures to a baseline of experiencing no visits and not visiting a facility.

	PP	aPD (95% CI)	aPR (95% CI)
No visit by CHV and no visit to facility	47.36	Ref	Ref
No visit by CHV and visit to facility	54.33	6.97 (2.31-11.63)	1.15 (1.05-1.25)
Visit by CHV and no visit to facility	48.43	1.07 (-5.51-7.66)	1.02 (0.88-1.16)
Visit by CHV and visit to facility	55.38	8.01 (0.43-15.61)	1.17 (1.01-1.33)

Abbreviations: PP, predicted prevalence, aPR, adjusted prevalence ratio; PD, adjusted prevalence difference; CI, confidence intervals; CHV, community health volunteer.

Table S11. Adjusted probabilities of unmet need for modern contraception by experience of household visits from a CHV and visit to a health facility. Prevalence ratio and prevalence difference compare different combinations of these exposures to a baseline of experiencing no visits and not visiting a facility.

	PP	PD (95% CI)	PR (95% CI)
No visit by CHV and no visit to facility	19.79	Ref	Ref

No visit by CHV and visit to facility	17.55	–2.24 (–6.66-2.18)	0.89 (0.66-1.11)
Visit by CHV and no visit to facility	15.89	–3.90 (–9.48-1.68)	0.80 (0.52-1.08)
Visit by CHV and visit to facility	14.00	–5.80 (–11.78-0.19)	0.71 (0.41-1.01)

Abbreviations: PP, predicted prevalence, PR, prevalence ratio; PD, prevalence difference; CI, confidence intervals; CHV, community health volunteer.
